# Brain-Targeted Delivery of Phenformin Using Phospholipid and Non-Phospholipid Vesicles for SHH Medulloblastoma

**DOI:** 10.3390/nano16090566

**Published:** 2026-05-04

**Authors:** Laura Di Magno, Federica Rinaldi, Luca Campea, Giorgia Della Rocca, Jacopo Forte, Eleonora D’Intino, Sara Cairoli, Bianca Maria Goffredo, Maria Carafa, Elena Del Favero, Carlotta Marianecci, Gianluca Canettieri

**Affiliations:** 1 Department of Molecular Medicine, Sapienza University of Rome, Viale Regina Elena 291, 00161 Rome, Italy; laura.dimagno@uniroma1.it (L.D.M.); luca.campea@uniroma1.it (L.C.); giorgia.dellarocca@uniroma1.it (G.D.R.); 2 Department of Drug Chemistry and Technology, Sapienza University of Rome, Piazzale A. Moro 5, 00185 Rome, Italy; federica.rinaldi@uniroma1.it (F.R.); eleonora.dintino@uniroma1.it (E.D.); maria.carafa@uniroma1.it (M.C.); 3 Department of Basic Biotechnological Sciences, Intensivological and Perioperative Clinics, Catholic University of Sacred Heart, Largo Francesco Vito 1, 00168 Rome, Italy; jacopo.forte@unicatt.it; 4 Division of Metabolic Diseases and Drug Biology, Bambino Gesù Children’s Hospital, IRCCS, Via Ferdinando Baldelli 38, 00146 Rome, Italy; sara.cairoli@opbg.net (S.C.); biancamaria.goffredo@opbg.net (B.M.G.); 5 Department of Medical Biotechnology and Translational Medicine, University of Milan, Via Fratelli Cervi 93, 20054 Segrate, Italy; elena.delfavero@unimi.it; 6 Institute Pasteur Italia, Fondazione Cenci-Bolognetti, Viale Regina Elena 291, 00161 Rome, Italy

**Keywords:** Hedgehog, phenformin, niosomes, medulloblastoma, drug targeting, biodistribution, nanocarriers, liposomes, brain drug delivery, blood-brain barrier

## Abstract

Medulloblastoma (MB) is the most frequent brain malignancy in children, frequently driven by deregulated Sonic Hedgehog (SHH) signaling. We previously identified the antidiabetic drug phenformin (Phen) as a potent Gli1 inhibitor that suppresses SHH-subtype MB growth. Despite its efficacy, systemic administration of Phen is limited by its potential to induce lactic acidosis, primarily through the suppression of hepatic gluconeogenesis. Here, we provide proof-of-concept that phospholipid (liposomes) and non-phospholipid (niosomes) vesicles (<200 nm) can be used to deliver phenformin selectively. Our results show that these vesicle-based delivery systems efficiently entrap Phen (around 50%) and release it into SHH MB cells, reducing proliferation and activating energy stress responses at higher doses. Furthermore, treated cells exhibit marked downregulation of SHH target genes Gli1 and Ptch1. In vivo, phenformin-loaded nanocarriers selectively increased drug accumulation in cerebellar tumors while minimizing systemic and hepatic exposure. Notably, niosomes demonstrated superior brain tumor targeting compared to free drug or liposome administration, as reflected by higher intratumoral concentrations of Phen compared to free drug or liposome administration. Consistent with this targeted delivery, we observed a substantial decline in intratumoral Gli1 and Ptch1 expression, confirming effective SHH pathway modulation. Together, these findings propose a promising nanotechnology-based method to improve phenformin therapeutic index in SHH MB by enhancing tumor specificity and reducing systemic toxicity.

## 1. Introduction

**Figure 1:**
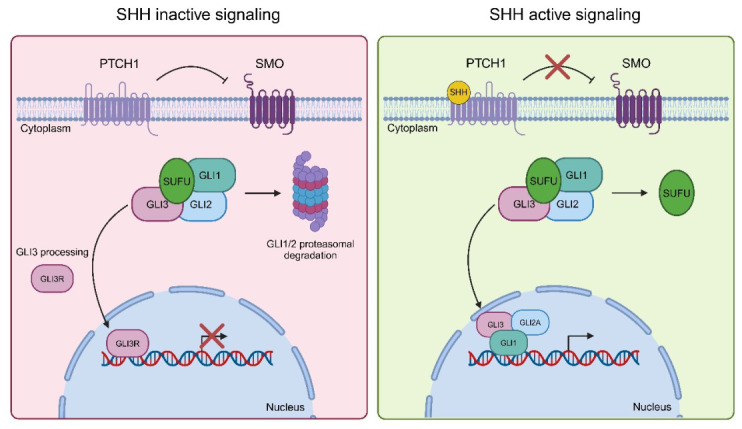
Sonic Hedgehog (SHH) signaling. Graphical illustration of inactive (left, red) and active (right, green) SHH pathways.

Medulloblastoma (MB) is the most frequent pediatric brain neoplasm and accounts for roughly 40% of all childhood tumors in the posterior fossa [1,2,3]. Recurrence is the most unfavorable prognostic factor for this tumor, occurring in approximately 30% of patients [4,5,6]. About 30% of all medulloblastomas and 60% of those occurring during infancy are associated with activating mutations of genes encoding components of the developmental Sonic Hedgehog (SHH) pathway. Activation of this pathway starts with the binding of the SHH polypeptide to the receptor Patched (PTCH), which subsequently alleviates the inhibition on the activator Smoothened (SMO). Upon activation, SMO triggers a cascade of events that culminates with the activation of the Gli transcription factors (Gli1, Gli2, and Gli3) (Figure 1, [7]).

The evidence that aberrant activation of the SHH signaling is associated with tumorigenesis has prompted efforts aimed at identifying strategies to target this pathway, leading to the identification and commercialization of two specific SMO inhibitors: Vismodegib and Sonidegib. Vismodegib received FDA approval in 2012 and Sonidegib in 2015, both for advanced basal cell carcinoma [8]. In medulloblastoma, their use remains investigational and subgroup-restricted: Phase I/II clinical trials demonstrated objective responses exclusively in patients with SHH-activated tumors, with no efficacy observed in non-SHH subgroups, establishing Hh pathway activation as a prerequisite biomarker for treatment eligibility [9,10,11,12]. Although these small molecules have shown a significant short-term efficacy when administered to SHH MB patients [13,14,15], their effectiveness is limited by the rapid development of drug resistance [16,17]. Two main mechanisms underlie this phenomenon: on-target resistance, arising from acquired point mutations within the SMO ligand-binding pocket that prevent drug binding while preserving downstream Hh signaling; and downstream resistance, occurring through amplification of GLI2 or CCND1, SUFU loss-of-function mutations, or aberrant activation of parallel oncogenic pathways such as PI3K/AKT and RAS/MAPK, all sustaining Hh transcriptional output independently of SMO inhibition [18,19,20]. Thus, alternative strategies bypassing the mechanisms of resistance are currently under investigation. MB cells are highly energy-demanding, with increased glucose metabolism and a strong susceptibility to nutrient deprivation. When the availability of nutrients is limited, the energy sensor AMPK phosphorylates the Gli1 transcription factor at three specific residues, promoting its ubiquitination by the E3 ligase β-TrCP and subsequent proteasomal degradation, inhibiting both GLI1 nuclear translocation and SHH transcriptional output [21,22]. Hence, AMPK activation plays a key role in the control of SHH MB cell proliferation, and AMPK agonists might be exploited in SHH MB therapy. Among the AMPK agonists, the antidiabetic drugs metformin and phenformin (Phen) are the best studied.

Both drugs share a common mechanism of action: inhibition of mitochondrial respiratory chain complex I, which reduces ATP synthesis, thereby triggering AMPK activation under conditions of energetic and metabolic stress [23]. Phenformin is approximately 50-fold more potent as a complex I inhibitor and, being more lipophilic and independent of organic cation transporter OCT1, achieves higher intracellular concentrations in tumor cells compared to metformin. Both metformin and Phen have been shown to possess significant antineoplastic properties against various types of cancers [24]. Phen is usually more effective than metformin against various cancer cell lines. Our recent studies have shown that Phen, unlike metformin, exhibits a strong antitumoral effect in preclinical models of SHH MB [25]. Phen is a positively charged molecule with more hydrophobicity and higher antitumor potency than metformin. However, Phen was withdrawn from clinical use due to the risk of lactic acidosis, as observed in diabetic patients treated with this drug [26]. Lactic acidosis results from mitochondrial respiratory chain inhibition, which blocks NADH oxidation mediated by complex I, causing NAD^+^ depletion and a rise in the NADH/NAD^+^ ratio. This redox imbalance drives the lactate dehydrogenase (LDHA) equilibrium toward pyruvate reduction to lactate, while simultaneously suppressing NAD^+^-dependent hepatic gluconeogenesis, impairing lactate clearance and further promoting lactate and H^+^ accumulation [27].

Therefore, one strategy to improve the safety profile of Phen with a reduction of its dangerous side effects would be its loading into drug delivery systems that allow the preferential release of the drug in tumor cells with minimal distribution of the drug in the liver or other organs. In addition to the well-known and already on the market liposomes, surfactant vesicles are increasingly being recognized as a valuable method to enhance drug delivery [28,29].

Niosomes are vesicular systems similar to liposomes, composed of synthetic surfactants combined with cholesterol or other amphiphilic compounds. They can form either unilamellar or multilamellar structures and are capable of encapsulating both hydrophilic and lipophilic agents, enabling targeted drug delivery. Surfactants and lipids used for niosomal and liposomal formulations are generally recognized as safe, and their production has lower costs. Additionally, surfactants exhibit greater stability under various conditions, thereby addressing some of the limitations associated with liposomes [30]. The overall features of these surfactant and phospholipid vesicles are specifically linked to their morphological properties such as size, shape, and surface chemistry.

Nanocarrier size and surface charge strongly influence BBB interaction and brain delivery. Nanocarriers in the 100–200 nm range show optimal adhesion to the endothelial membrane, while larger liposomes (>300 nm) are more affected by hemodynamic stress, reducing brain deposition [31,32]. Surface charge also plays a key role: cationic nanocarriers (+20 to +40 mV) achieve significantly higher brain accumulation (up to 2–3 times more than anionic nanocarriers (−10 to −30 mV) or neutral nanocarriers (~0 mV) [28]). Anionic liposomes still interact with the cell surface more than neutral ones but result in lower deposition in brain tissue. Optimizing both size and charge is therefore crucial for efficient BBB targeting. A deep physical–chemical characterization is fundamental and necessary to understand nanocarrier features, as they significantly impact the drug’s inherent pharmacokinetics and its targeting to pathological areas.

The aim of this study was to develop phenformin-loaded liposomes and niosomes and evaluate their physicochemical properties, cellular activity, and in vivo tumor targeting in SHH medulloblastoma models. We demonstrated that Phen-loaded niosomes and liposomes are well suited to efficiently release the drug in MB cells, reducing their proliferation and most relevantly, they may reach intracranial SHH MB tumors in animal models, crossing the blood–brain barrier (BBB) and leading to significantly increased tumor delivery and reduced systemic and hepatic concentrations.

## 2. Materials and Methods

### 2.1. Chemicals and Reagents

1,2-Dimyristoyl-sn-glycero-3-phosphocholine (DMPC, purity > 99%) was from Avanti Polar Lipids (Alabaster, AL, USA). Polyoxyethylene (20) sorbitan monolaurate (#93774), Tween 20 (Tw20, #P9416, assay > 40% (GC)), cholesterol (Chol, #C8667, purity ≥ 99%), Hepes salt {N-(2-idroxyethyl) piperazine-N′-(2 ethanesulfonicacid)} (#H3375, ≥99.5% (titration)), pyrene (#571245, sublimed grade, 99%), 1,6-Diphenyl-1,3,5-hexatriene (DPH, #D208000, 98%), calcein (#C0875), Nile Red (#72485), human serum (HS, #H4522), bovine serum (BS, #B8655), and phenformin hydrochloride (Phen, #P7045) were from Sigma-Aldrich (St. Louis, MO, USA). The artificial cerebrospinal fluid (aCS, #3525) was from Tocris Bioscience (Bristol, UK).

### 2.2. Preparation and Purification of Vesicles

Phen-loaded surfactants (NVs) and phospholipid vesicles (LVs) were prepared with various proportions of Tween 20, DMPC, and cholesterol (Chol) (Table 1). The concentration of Tween 20 consistently exceeded the critical micelle concentration (CMC) of 0.048 mM in water at 20 °C [33]. Unilamellar phospholipid and non-phospholipid vesicles were produced using the “film” method [34], followed by sonication at 25 °C or 60 °C, respectively, for 15 min at an amplitude of 18%. The samples were sonicated using a 500 W ultrasonic processor (Vibracell-VCX 500, Sonics, Taunton, MA, USA) equipped with a titanium tapered microtip designed for 500–750 W ultra-high-intensity applications. The microtip had a diameter of 3 mm and was suitable for sample volumes of 1–10 mL. To prevent overheating during sonication, the samples were kept in a bath (25 °C for LVs and 60 °C for NVs). The dried films were rehydrated using different aqueous phases: a Phen solution (41 mM) and a sodium calcein solution (10^−2^ mM), both in Hepes buffer (10 mM, pH 7.4) for cell interaction studies. Nile Red, a lipophilic fluorescent probe, was loaded (final concentration 0.79 mg/mL) during the film formation by adding the components and then by solubilizing with organic solvents. The resulting vesicles were subsequently purified via centrifugation (25 °C, 3000 rpm, 20 min) using an MPW-260R centrifuge (MPW Med. Instruments, Warsaw, Poland). All studies were carried out on purified vesicles.

### 2.3. Physical Characterization

#### 2.3.1. Dynamic Light Scattering (DLS)

DLS analyses were carried out on NVs and LVs, both with and without Phen, to determine the average diameters (Z-Average), ζ-potential, and size distributions (polydispersity index, PDI) at 25 °C. These measurements were performed on a Zetasizer Nano ZS 90 (Malvern Panalytical, Malvern, UK), with a scattering angle of 90.0° and equipped with a 5 mW HeNe laser (λ = 632.8 nm) and a digital logarithmic correlator. Electrophoretic mobility measurements were performed using the laser Doppler electrophoresis technique with the same Malvern Zetasizer Nano ZS 90 apparatus. ζ-potential was calculated by the Smoluchowski relation, ζ = uη/є, with η = viscosity and є = permittivity of the solvent phase [35,36]. All the measurements were performed on each sample at 1:10 dilution in the appropriate Hepes buffer. The reported particle size and PDI values represent the average of three independent measurements for each sample. Regarding stability evaluation criteria, particle size variations within 5–10% of the initial measurement (essential to remain below the 200 nm threshold for BBB delivery) and PDI values remaining below 0.2 were considered acceptable, with no significant aggregation observed during the stability evaluation. These characteristics are expected to favor circulation and tissue retention while minimizing clearance, supporting effective BBB targeting.

#### 2.3.2. Small Angle X-Ray Scattering (SAXS)

SAXS measurements were performed at the ID02 beamline of the European Synchrotron Radiation Facility (ESRF) in Grenoble, France [37]. Scattered intensities were collected within the range 0.08 nm^−1^ < q < 4 nm^−1^, where q represents the scattering vector defined as q = 4π sin(θ/2)/λ, with θ being the scattering angle and λ = 0.1 nm the wavelength of the incident radiation. Following background subtraction, the resulting intensity profiles provided insights into the internal structure of the vesicles at the nanometer scale. The intensity profiles of lipid vesicles show a series of bumps (absent for a homogeneous sphere), accounting for the vesicles’ shell form factor, headgroups–tails–headgroups [38]. In multilamellar systems, additional equally spaced intensity peaks are visible at q1 = 2π/d, q2 = 2* 2π/d, and qi = i* 2π/d, where d is the interlamellar distance, accounting for both the thickness of the lipid bilayer and the water interlamellar layer. In phospholipid–cholesterol-based vesicles, q1 = 1 nm^−1^, corresponding to d = 6 nm, of which 4–5 nm are due to the thickness of the bilayer [39].

#### 2.3.3. Physical Stability

Colloidal stability at different temperatures was evaluated on both samples (Phen-loaded NVs and LVs) when stored at 4 and 25 °C for a period of 90 days. Samples from each batch were withdrawn at definite time intervals (1, 30, 60 and 90 days) and both the mean hydrodynamic diameter and the ζ-potential were evaluated as previously described. To examine the biological stability of Ph-NVs and Ph-LVs, similar analyses were performed with human serum (HS) and artificial cerebrospinal fluid (aCSF). Specifically, vesicle suspensions were supplemented with proper amounts of HS or aCSF until reaching concentrations of 45% or 90%, respectively.

#### 2.3.4. Bilayer Characterization

Fluorescence experiments were performed on Ph-NVs and Ph-LVs containing pyrene and DPH using a LS55 spectrofluorometer (PerkinElmer, Waltham, MA, USA) to assess the microviscosity, polarity, and fluidity of the hydrophobic bilayer within the vesicles. Pyrene facilitates the analysis of the lateral distribution and movement of compounds within the membrane. At high concentrations, pyrene typically forms excimers. A decrease in excimer fluorescence corresponds to an elevation of monomer fluorescence. The pyrene monomer fluorescence spectrum consists of five peaks. The ratio of the intensities of the first (I1) and third (I3) vibrational bands (I1/I3) indicates the polarity of the pyrene environment [40]. A low I1/I3 ratio indicates a nonpolar environment; higher values are related to an increase in medium polarity [41]. Since pyrene is localized inside the hydrophobic bilayer of the vesicles, its fluorescent behavior in our systems refers to the bilayer characteristics [42]. Pyrene may form intramolecular excimers. Therefore, the IE/IM ratio, where IM represents monomer fluorescence intensity and IE represents excimer fluorescence intensity, is utilized to evaluate microviscosity. Additionally, the pyrene probe can qualitatively indicate changes in micropolarity within the solubilization region by observing variations in the ratio of monomer vibronic band intensities at 377 nm and 397 nm [43]. The fluidity of the Ph-LVs and Ph-NVs bilayers was evaluated using fluorescence anisotropy analyses with DPH as the probe. DPH was integrated into the vesicles at a final concentration of 2 × 10^−3^ M. DPH stock solution was prepared to achieve a final concentration of 2 × 10^−3^ M. To produce DPH-labeled vesicles, 200 μL of the stock solution was mixed with the solubilized components in the organic mixture, and the vesicles were formed using the thin film evaporation method as previously described.

#### 2.3.5. Phenformin Entrapment Efficiency and In Vitro Drug Release

UV-vis spectroscopy has been employed to evaluate the amount of Phen entrapped in NVs and LVs and its release by in vitro experiments. Initially, a calibration curve of phenformin was obtained by measuring the UV absorbance at 240 nm of a series of standard Phen solutions at known concentrations dissolved in a mixture of ethanol: Hepes 30:70 (*v*/*v*), using a UV-vis spectrophotometer (Lambda 25, PerkinElmer, Waltham, MA, USA). The analytical calibration curve showed a linear correlation between absorbance and concentration and was subsequently used to determine Phen concentration in loaded NVs and LVs. E. E. % was calculated as:

$$E.\;E.\;\%=\frac{Entrapped\;drug\;\left(mg\right)}{Total\;drug\;used\;\left(mg\right)}\times 100$$

Phen release studies from drug-loaded NVs and LVs were performed using dialysis tubes (MW cut-off 8000 and 5.5 cm^2^ diffusing area) at 37 °C in 90% aCSF. The release medium (ethanol: Hepes 30:70 *v*/*v*) volume was chosen to maintain sink conditions, ensuring that Phen concentration in the external medium remained well below its solubility limit throughout the experiment. A temperature-controlled water bath maintained the setup at 37 °C, with the release medium magnetically stirred throughout. At established intervals, 1 mL of the samples was collected for UV analysis and promptly returned to the external medium. Phen release was quantified using a Perkin-Elmer Lambda 3a UV-vis spectrometer. The percentage of Phen released was calculated by comparing the cumulative amount of Phen detected in the release medium with the total amount of Phen initially encapsulated in NVs or LVs, according to the following equation:

% of Phen released = (Amount of Phen detected in release medium/Total Phen entrapped in nanocarrier) × 100

All release experiments were performed in triplicate, and the reported values represent the mean, with deviations within 10% of the mean.

### 2.4. Biological Characterization

#### 2.4.1. Cell Lines and Cultures

The mouse Med1-MB cell line was donated by Dr. Yoon-Jae Cho, Stanford University School of Medicine (Stanford, CA, USA) [44], authenticated by short tandem repeat (STR) profiling, and routinely tested for mycoplasma contamination using a PCR-based detection kit (#G238, abm); all cells used in this study were mycoplasma-free. Med1-MB cells were grown in Dulbecco’s modified Eagle medium (DMEM, #6546, Sigma-Aldrich) containing 10% fetal bovine serum (#A5256701, GIBCO), 1 mM penicillin–streptomycin (#P0781, Sigma-Aldrich), and 1 mM L-glutamine (#G7513, Sigma-Aldrich). The human DAOY cell line was purchased from ATCC (#ATCC- HTB-186, Rockville, VA, USA) and was cultured as previously described [22]. For Smoothened agonist (SAG) treatments, DAOY cells were cultured overnight in serum-free medium supplemented with 1% bovine serum albumin (BSA), followed by incubation with SAG (200 nM) for 36 h. For Phen treatments, cells were incubated with free Phen or Ph-NVs in DMEM without glucose and sodium pyruvate (#11966-025, GIBCO, Thermo Fisher Scientific, Waltham, MA, USA) with 10% FBS, 1 mM penicillin–streptomycin, 1 mM L-glutamine and 0.75 mM glucose (D-(+)-Glucose solution, #G8644, Sigma-Aldrich). Glucose concentration reflects the estimated physiological glucose level in the tumor tissue microenvironment [25,45].

#### 2.4.2. Immunofluorescence

Cells were seeded on 8-well coverslips and treated with free NVs, LVs or Ph-NVs, Ph-LVs or Nile Red-loaded Ph-NVs, or Ph-LVs for 6 h. Cells were then fixed and evaluated under a fluorescence microscope (Leica DM2500, Leica Microsystems, Wetzlar, Germany) equipped with a 40× objective. Nuclei were revealed using bisBenzimide H 33342 trihydrochloride (Hoechst—10 μg/mL, Sigma-Aldrich #14533) staining and visualized using a DAPI filter set (excitation 360 nm/emission 460 nm). Nile Red fluorescence was detected using a TRITC filter set (excitation 541 nm/emission 572 nm). Images were acquired using a Leica DFC camera and analyzed using ImageJ software (version 1.54, National Institutes of Health, Bethesda, MD, USA).

#### 2.4.3. Western Blot

Med1-MB cells were plated in a 12-well dish and exposed to free Phen, Ph-NVs, or Ph-LVs for 2 h. After treatments, cells were lysed, total proteins were separated on an 8% Bis-Tris polyacrylamide gel, and transferred to a nitrocellulose membrane (#1215471, GVS). Membranes were incubated with 5% nonfat dried milk in PBS buffer (blocking solution) containing 0.1% Tween20 (PBST; pH 7.2) and incubated with primary antibodies overnight. Filters were incubated with secondary antibodies (phospho-ACC (#3661 Cell Signaling Technology, Danvers, MA, USA, WB 1:1000), ACC (#3662 Cell Signaling Technology, WB 1:1000), and Vinculin (#sc-73614 SantaCruz Biotechnology, Dallas, TX, USA, WB 1:1000) and visualized using enhanced chemiluminescence (WesternBright ECL, #K-12045-D50, Advansta, San Jose, CA, USA).

#### 2.4.4. Proliferation Assay

In this assay, 2 × 104 Med1-MB cells and 2 × 104 DAOY cells were seeded in 12-well plates and exposed to either free Phen, Ph-NVs, or Ph-LVs at a concentration of 5 µM. After 48 h, cells were trypsinized (Trypsin-EDTA, #T4049 Sigma-Aldrich) and counted by the Trypan Blue (#93595, Sigma-Aldrich) exclusion method.

#### 2.4.5. IC50 Calculation

For IC50 determination, Med1-MB cells were treated with five scalar concentrations of free Phen or Ph-NVs or Ph-LVs (range: 0–1000 μM) over a 24 h period, each tested in technical triplicate and repeated in at least three independent biological experiments. Cell viability data were normalized to vehicle-treated controls and expressed as proliferation (%). IC50 values were calculated by nonlinear regression using a four-parameter dose–response curve fitting model (GraphPad Prism v.6.0, GraphPad Software, La Jolla, CA, USA).

#### 2.4.6. Quantitative Real-Time PCR (qRT-PCR)

Total mRNA was extracted from cells and reverse transcribed with the Sensifast cDNA synthesis kit (#BIO-65054, Bioline, London, UK). Quantitative PCR was carried out using the SensiFast Sybr Lo-Rox Mix (#BIO-94020, Bioline, London, UK). Primer specificity was confirmed by melt curve analysis, which showed a single dissociation peak for each amplicon. Primer amplification efficiency was determined by serial dilution of cDNA and calculated from the slope of the resulting standard curve. Only primers with amplification efficiency more than 90% were used in this study. L32 mRNA levels were measured to normalize each sample [46,47]. Primer sequences are provided below:

mGli1: forward GCCAACTTTATGTCAGGGTCCCAG; reverse GGAGAGAGCCCGCTTCTTTGTTAA;

mPtch1: forward TGACAAAGCCGACTACATGC; reverse AGCGTACTCGATGGGCTCT;

mL32: forward AGAGGTGCTGGGAGCTGCTA; reverse GATGGATGGTCTCTGGACGG;

hGli1: forward GGGATGATCCCACATCCTCAGTC; reverse CTGGAGCAGCCCCCCCAGT;

hPtch1: forward CGATGGAGTCCTTGCCTACAA; reverse CCACCAGACGCTGTTTAGTCA;

hL32: forward CCCTGGTGAAGCCCAAGATC; reverse TCTGGGTTTCCGCCAGTTAC.

#### 2.4.7. In Vivo Antitumor Efficacy

Math1-Cre/Ptch1^LoxP-LoxP^ mice were a gift from Dr. Robert Wechsler-Reya (Duke University Medical Center, Durham, NC, USA) and were previously described [25]. The animals were fed with a standard chow diet at a temperature of 23 °C. Mice were randomly divided into four groups, each comprising five mice: Group 1 (NT, control group), Group 2 (100 mg/kg of free Phen), Group 3 (Ph-LVs equivalent to 100 mg/kg of Phen), Group 4 (Ph-NVs equivalent to 100 mg/kg of Phen). The preparations were delivered to the mice via intravenous injection into the tail vein at a volume of 0.1 mL/10 gr body weight. After 24 h, blood samples were collected and immediately centrifuged at 2500 *g* for 5 min to obtain plasma. Mice were anesthetized with Zoletil (50 mg/Kg, Virbac S.r.l.) + Rompun (10 mg/kg, Elanco Italia S.p.A) by intraperitoneal injection and then euthanized by cervical dislocation. At the end of the treatment, total mean weight was 21.4 gr ± 1.4 gr; individual weights were: Group 1 = 19.5 g, 20.4 g, 21.5 g, 22.3 g, 23.2 g; Group 2 = 19.8 g, 21.5 g, 21.6 g, 23.2 g, 19.9 g; Group 3 = 19.5 g, 20.3 g, 21.7 g, 22.8 g, 23 g; Group 4 = 23.1 g, 19.9 g, 19.6 g, 22.9 g, 22.3 g. Tissues (i.e., MB and liver) were also excised from all the mice and collected. Determination of Phen concentration was performed as previously described [25]. All animals were maintained according to the guidelines set out in Commission Recommendation 2007/526/EC. Animal procedures were approved by the Animal Ethics Committee of the Department of Molecular Medicine—Sapienza University of Rome and conducted in accordance with Italian Governing Law for animal welfare (D.L. 26/2014). The experiments were approved by the Italian Ministry of Health (Protocol Number: 03/2013).

#### 2.4.8. Statistical Analysis

GraphPad Software (v.8, La Jolla, CA, USA) was used to perform statistical analysis. Data are expressed as mean ± SD. Sample sizes are indicated in the figure legends. A two-way ANOVA was performed to assess the combined effects of two independent variables on the measured parameters, followed by Tuckey’s multiple comparisons test or by Sidak’s multiple comparison tests. Results were considered statistically significant at (*p* < 0.05, *), (*p* < 0.01, **), (*p* < 0.005, ***) and (*p* < 0.0001, ****). No data were excluded from the analyses. All experiments were performed at least three independent times unless otherwise stated.

## 3. Results

### 3.1. Physical Characterization

#### 3.1.1. Dynamic Light Scattering (DLS) Measurements

Niosomes and liposomes are amphiphilic-based vesicles composed of surfactants and phospholipids together with cholesterol, which was incorporated into the preparation to enhance the cohesion of the nonpolar regions of the vesicular bilayer. After loading Phen in the two vesicles, we performed dynamic light-scattering analyses on purified samples. Phen-loaded niosomes (Ph-NVs) show an increased size (191 nm) in comparison with lipid vesicles (Ph-LVs) (160 nm), and the size of empty vesicles is comparable with those loaded with Phen. Furthermore, the liposomal formulation exhibits a less negative ζ-potential than niosomal vesicles, along with a higher PDI value, suggesting lower vesicle homogeneity. However, since PDI remains below 0.3, the formulation can still be considered fairly homogeneous, although to a lesser extent than niosomes (Table 2). Based on the obtained data, the presence of Phen in the preparation does not seem to change the size and ζ-potential. Therefore, in accordance with its solubility characteristics, the drug should be included in the aqueous core of the vesicles. These results are consistent with the data from Pereira-Nunes et al. [48]. Although the two systems differ in size, these variations are not statistically significant and are unlikely to result in meaningful differences in their interaction with biological membranes. No statistically significant differences were detected between LVs and Ph-LVs, nor between NVs and Ph-NVs, indicating that the inclusion of Phen did not produce significant changes within each corresponding sample (*p* > 0.05). In contrast, statistically significant differences were observed when comparing the two different sample types, namely LVs versus NVs and Ph-LVs versus Ph-NVs (*p* < 0.001), highlighting a clear distinction between these samples regardless of Phen’s presence.

**Figure 2:**
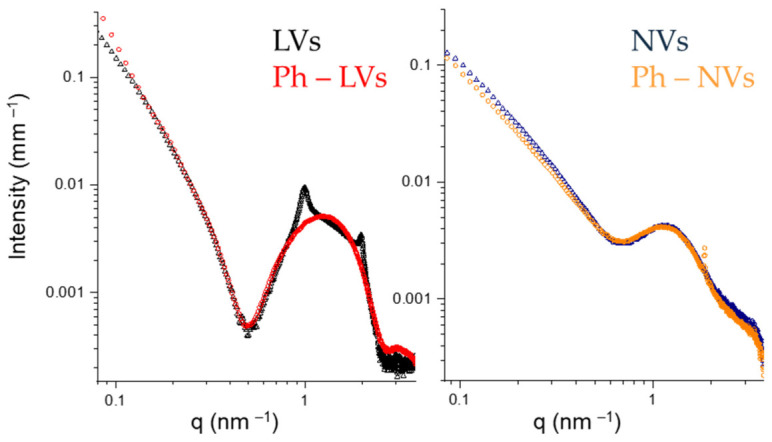
SAXS intensity profiles of vesicles at 20 °C: LVs (black), Ph-LVs (red), NVs (blue), Ph-NVs (orange).

UV analyses revealed that Phen entrapment efficiency (E.E. %) was approximately 50% at a drug concentration of 10 mg/mL in the hydrating solution (Table 2). Given this entrapment efficiency, the amount of Phen encapsulated within the vesicular system is expected to be pharmacologically sufficient to elicit the intended therapeutic effect [25]. Indeed, Di Magno et al. demonstrated the antitumor efficacy of Phen following tail vein administration at doses ranging from 1 to 12.5 mg/kg. The implementation of a drug delivery system may enable dose reduction of Phen while enhancing its selective accumulation and controlled release at the target site compared with administration of the free drug. The internal structure of the systems was investigated by SAXS. The intensity profiles reported in Figure 2 show common features, a first pronounced minimum and a hump, that are characteristic of vesicles with a layered shell. In liposomes, several lipid bilayers, each one about 5 nm thick, are stacked in a multilayered shell, with a 6 nm interlamellar distance. The presence of Phen does not modify the overall structure of LVs and the bilayer thickness. Rather, the intensity peaks due to the multilamellar structure of the shell disappear, indicating the formation of unilamellar vesicles. This result suggests the interaction of Phen with the phospholipid headgroups, favoring a spontaneous curvature of the lipid bilayer. In niosomes, a single surfactant-based bilayer is seen in both NVs and Ph-NVs. The intensity profiles of the two systems are perfectly superimposed, revealing that Phen did not change the packing of Tween and cholesterol.

#### 3.1.2. Physical Stability

**Figure 3:**
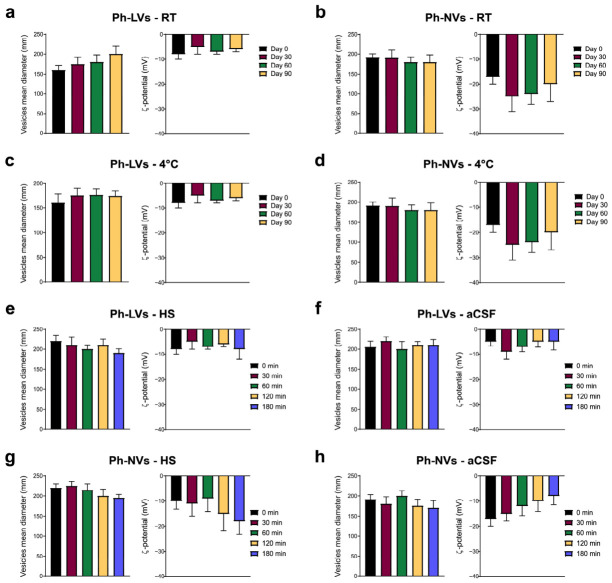
Stability of phenformin-loaded vesicles (Ph-LVs and Ph-NVs). (**a**,**b**) Shelf stability analysis of Ph-LVs (**a**) and Ph-NVs (**b**), focusing on changes in hydrodynamic diameter and ζ-potential over a period of 90 days at room temperature (RT). (**c**,**d**) Assessment of shelf stability of Ph-LVs (**c**) and PH-NVs (**d**), considering variations in hydrodynamic diameter and ζ-potential over 90 days at 4 °C. (**e**,**f**) Evaluation of Ph-LVs’ biological stability with HS (**e**) and aCSF (**f**) for up to 3 h at 37 °C. (**g**,**h**) Evaluation of Ph-NVs’ biological stability with HS (**g**) and aCSF (h) for up to 3 h at 37 °C. Data are presented as mean ± SD (*n* = 3).

Shelf stability studies showed that Ph-NVs has a higher colloidal stability when stored at 25 °C and 4 °C in a time interval of 90 days, in terms of dimension and ζ-potential variations (Figure 3a–d). Ph-LVs showed slight variations both in dimensions and ζ-potential, due to the non-homogeneous sample at the starting point. Phen-loaded liposomal and niosomal dispersion stability was also evaluated at 37 °C in HS and aCSF. Ph-NVs and Ph-LVs resulted in immediate coating by HS and aCSF proteins with a slight dimension increase but no significant changes in ζ-potential over 180 min (Figure 3e–h). As reported by Van Straten et al., the aCSF coating occurs after just one hour of interaction, so we did not extend the interaction study [49].

#### 3.1.3. Bilayer Characterization

The membrane fluidity was investigated by loading the DPH “fluidity probe” into Ph-NVs and Ph-LVs, as previously described [40]. The fluorescence anisotropy data indicates that the vesicular bilayer is quite fluid and that the obtained values are similar for both systems (Table 3). Phen loading increases membrane fluidity, probably because of the partial interaction of Phen with the phospholipid headgroups, favoring a spontaneous curvature of the lipid bilayer in liposomes and producing only a more fluid bilayer in niosomal vesicles (the anisotropy value for both unloaded vesicles is around 0.25) [16]. To gain deeper insights into the bilayer characteristics, we assessed its microviscosity and polarity (Table 3). Both values are similar for both preparations with Phen. The presence of Phen in both vesicular structures does not affect polarity (polarity of free LVs and NVs is 1.1), while microviscosity is increased compared to that of free vesicles (the microviscosity of free LVs and NVs is 0.4). Probably, microviscosity is increased because of Phen interaction with hydrophilic heads of amphiphilic compounds but does not affect polarity because water does not penetrate the bilayer. Summarizing, bilayer characterization indicates that the addition of Phen increases bilayer fluidity and viscosity without altering its polarity.

#### 3.1.4. Phenformin Release Studies

**Figure 4:**
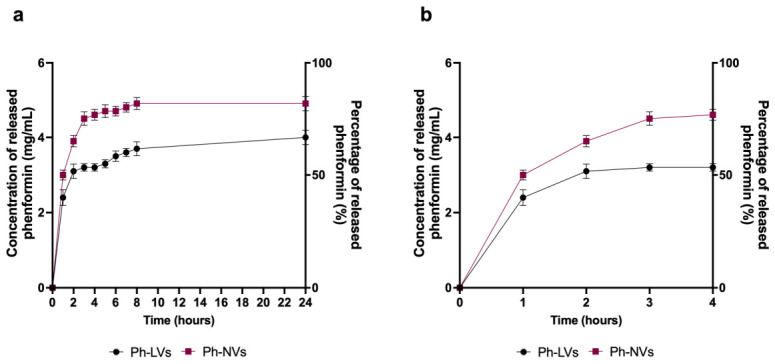
Release profile of phenformin from Ph-LVs and Ph-NVs. Phenformin release profiles of Ph-LVs (**a**) and Ph-NVs (**b**) were evaluated in aCSF. Panel a shows the release measured at 24 h, while panel b shows the release measured at 4 h. Data are represented as means ± SD (*n* = 3).

Release studies indicate that Phen release in aCSF is comparable for liposomal and niosomal vesicles and occurs rapidly, with a substantial amount released (approximately 50% after 2 h; Figure 4a shows the 24 h profile, while Figure 4b highlights the first 4 h). The release percentage obtained at 4 h increases slightly over 24 h, demonstrating that in aCSF the vesicles are able to release the drug in large quantities after only 4 h. This behavior, although more evident for niosomal vesicles, could be related to the high fluidity characteristics of both systems (Table 3) [50].

### 3.2. Intracellular Delivery of Phenformin-Loaded Vesicles in SHH MB Cells

**Figure 5:**
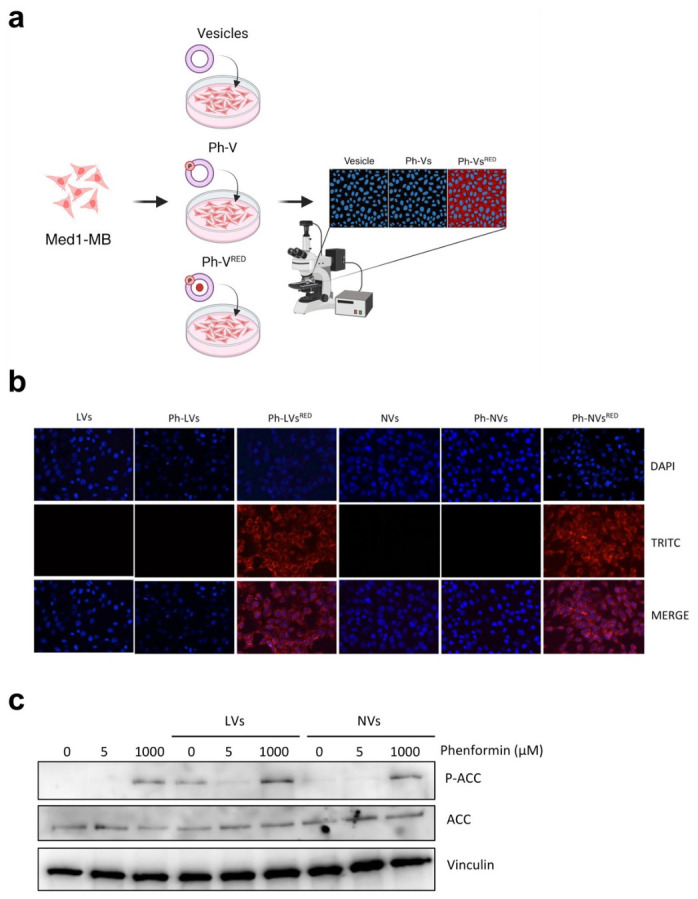
Intracellular delivery of phenformin in medulloblastoma cells. (**a**) Schematic overview. (**b**) Uptake of Phen-loaded vesicles as shown by fluorescent microscopy in Med1-MB cells after 6 h of incubation. Vesicles contained Nile Red (red), and Hoechst was used to stain cells. (**c**) Western blot analysis of Med1-MB cells treated as indicated for 2 h. Cell extracts were immunoblotted as indicated in the figure. Vinculin was used as a loading control. Raw images of the blots are shown in Figure S1.

We next investigated the efficacy of the two different vesicular formulations in delivering Phen in Med1-MB cells, a primary tumor cell line of SHH MB [44,51,52], where the antitumor effects of Phen were previously demonstrated [25]. These cells are derived from an MB arising in a Ptch1+/−; lacZ mouse [44] and, unlike primary SHH MB cells, maintain elevated Gli1 expression over multiple passages and show sustained Hh pathway activation. This activation is mediated via SMO and can be suppressed by Smo inhibitors (e.g., KAAD-cyclopamine) or Gli antagonists [53]. We incubated Med1-MB cells with empty or Phen-loaded LVs or NVs, co-loaded with Nile Red or unlabeled. Cellular uptake was analyzed under a fluorescent microscope (Figure 5a). Both vehicles delivered Phen into Med1-MB cells at comparable efficacy, as determined by the levels of red staining for the two formulations (Figure 5b). To further confirm the ability to mediate the delivery of Phen in MB cells, we treated Med1-MB cells with free Phen or Ph-NVs at two different doses: 5 µM, corresponding to the maximum tolerated circulating concentration in mice, and 1 mM, a mitochondrial complex I inhibitory dose [25]. We analyzed the functional status of AMPK, a well-known target of biguanides, activated by increased AMP levels following complex I inhibition [54], by measuring the phosphorylation levels of its substrate ACC. Consistent with our previous results, 5 µM of free or encapsulated Phen did not change the phosphorylation status of ACC, which was instead significantly increased when cells were treated with 1 mM of Ph-NVs or Ph-LVs, achieving an approximate 3-fold increase relative to the control (Figure 5c), demonstrating that both formulations were equally effective in delivering the drug into the cells.

### 3.3. Phenformin-Loaded Vesicles Inhibit SHH MB Cell Proliferation and SHH Signaling

**Figure 6:**
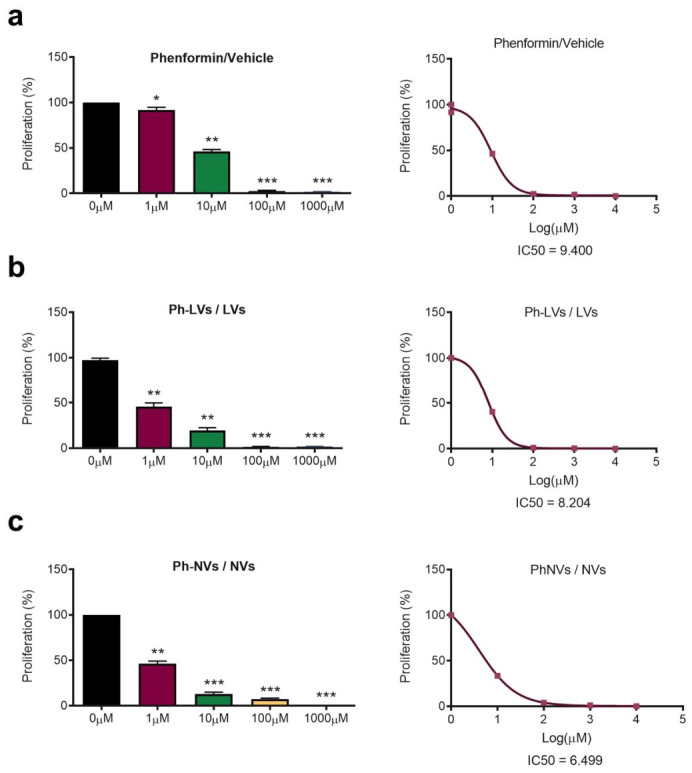
Antiproliferative efficacy of phenformin-loaded vesicles in medulloblastoma cells. (**a**) (Left) Proliferation rate of Med1-MB cells exposed to varying doses of Phen for 24 h. IC50 values of Phen were established using nonlinear regression (right). (**b**) (Left) Med1-MB cells were incubated with the indicated doses of either free LVs or Ph-LVs for 24 h, and cell proliferation was assessed at the end of the treatment. (Right) IC50 values are represented. (**c**) (Left) Proliferation assay performed on Med1-MB cells exposed to free NVs or Ph-NVs at specified concentrations for 24 h. (Right) IC50 values were calculated for both formulations. Data are expressed as mean ± SD from three independent experiments, each conducted in triplicate. * *p* < 0.05, ** *p* < 0.01, *** *p* < 0.001 as determined by ANOVA test.

We next evaluated the antiproliferative activity of vesicular formulations on Med1-MB cells by treating the cells with increasing concentrations of Phen for 24 h and measuring the number of viable cells by the Trypan Blue exclusion method. The results showed a dose-dependent decrease in cell number, with an IC50 value in the low μM range, making NVs more active than LVs or free Phen (Figure 6).

**Figure 7:**
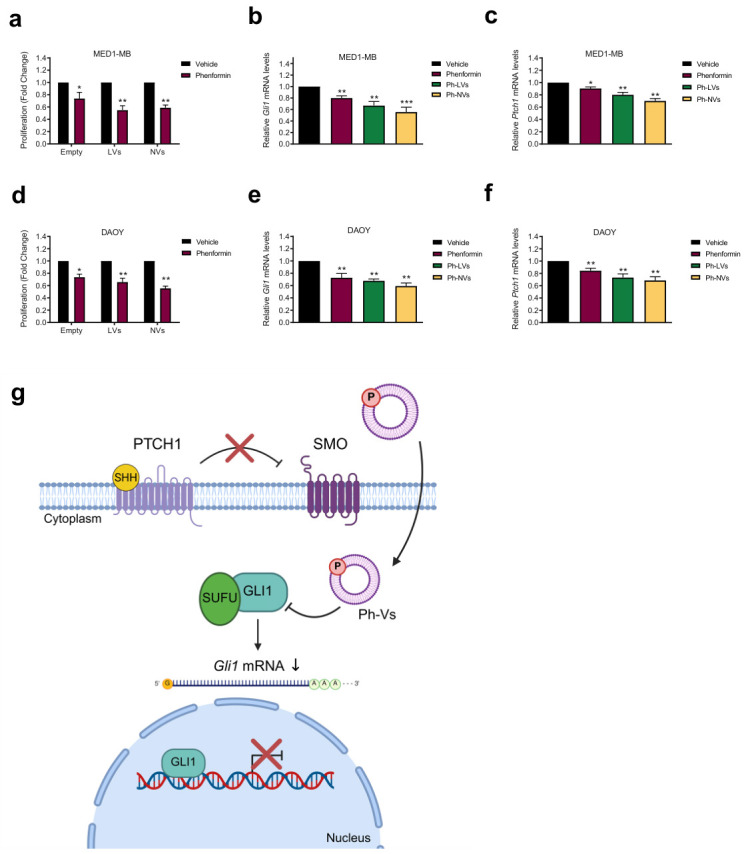
Effect of phenformin-loaded vesicles on Hh-dependent proliferation. (**a**) Growth assay in Med1-MB cells treated with 5 µM Phen, or Ph-NVs or Ph-LVs for 48 h. (**b**,**c**) Expression levels of *Gli1* or *Ptch1* mRNA were determined by qPCR. Values were normalized over *L32* mRNA expression levels. (**d**) Proliferation assay in SAG-treated DAOY cells after incubation with 5 µM Phen, or Ph-NVs or Ph-LVs for 48 h. (**e**,**f**) *Gli1* and *Ptch1* mRNA expression levels were quantified by qPCR, and transcript levels were normalized relative to *L32* mRNA expression. (**g**) Schematic representation of the mechanism of action of Phen-loaded vesicles in cellular models. Statistical significance was measured using ANOVA: * *p* < 0.05, ** *p* < 0.01, *** *p* < 0.001.

Since in our previous work we found that therapeutic doses of Phen limit SHH MB cell proliferation by inhibiting Hh-Gli1 signaling, we next investigated the ability of the different vesicular formulations to inhibit the proliferation rate of Hh-dependent MB cells. We treated Med1-MB cells with free Phen or Ph- NVs or Ph-LVs at the clinically relevant doses (5 μM) and analyzed cell proliferation and *Gli1* mRNA levels. The results show a significant antiproliferative effect of free and vehiculated Phen, associated with the inhibition of Hh signaling (Figure 7a–c). Nanoencapsulation enhances the antiproliferative activity of Phen, providing Ph-LVs and Ph-NVs a similar efficacy, resulting in an approximate 40% reduction in cell proliferation (Figure 7a). Both formulations inhibited *Gli1* and *Ptch1* expression with higher efficacy than Phen alone, with the effect of Ph-NVs more pronounced than that of Ph-LVs (Figure 7b,c) and confirming the ability of intracellular Phen to prevent Hh activity (Figure 7g). Similarly, inhibition of cell proliferation (approximate 45% reduction) and *Gli1* and *Ptch1* mRNA expression was observed in Hh-induced human MB DAOY cells (Figure 7d–f), suggesting that the effect is generalizable to other SHH-MB subtypes.

### 3.4. Biodistribution and Hedgehog Pathway Inhibition After Delivery of Ph-Loaded Vesicles in Animal Models of SHH MB

**Figure 8:**
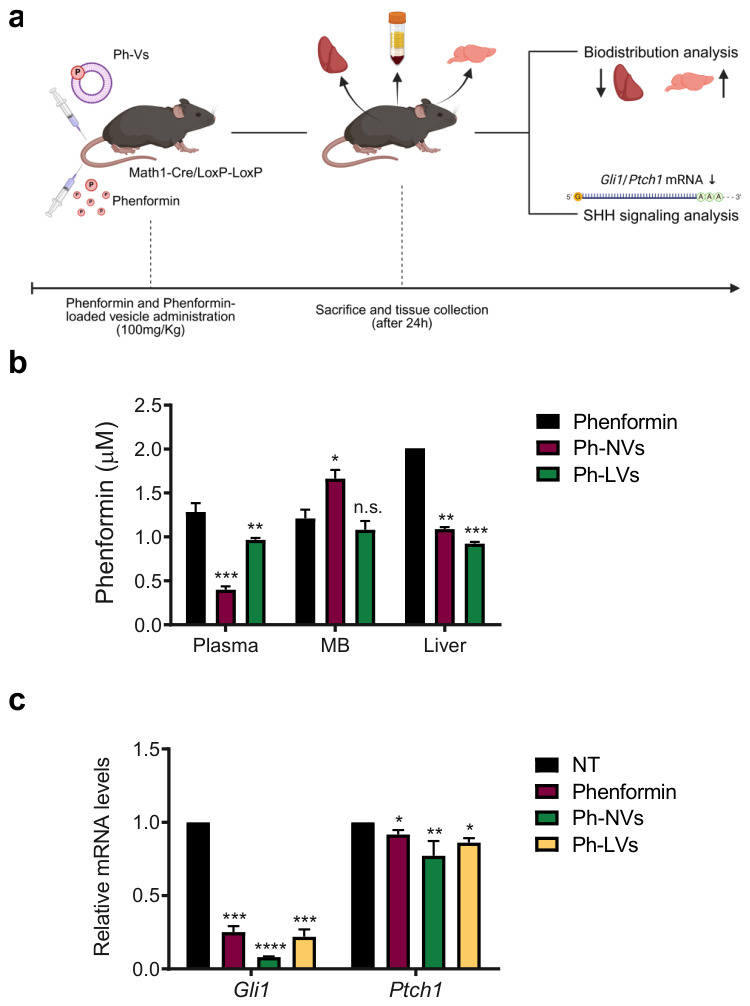
Biodistribution of phenformin-loaded vesicles and Hh pathway inhibition. (**a**) Scheme of the experimental design in MB mouse models. Arrows indicate decreased (downward) and increased (upward) levels. (**b**) Tissue concentration distribution profile of free Phen, Ph-NVs or Ph-LVs after intravenous administration at a dose of 100 mg/kg. (**c**) Expression levels of *Gli1* and *Ptch1* mRNA in MB tissue, as determined by qPCR. Data were normalized to *L32* expression levels. Statistical significance was determined using ANOVA: * *p* < 0.05, ** *p* < 0.01, *** *p* < 0.001, **** *p* < 0.0001, n.s. = not significant.

To determine the biodistribution of these nanocarriers and their ability to inhibit the activity of the Hh pathway in vivo, we intravenously administered vesicles in SHH MB mouse models (Figure 8a). A total of 100 mg/Kg Phen-loaded vesicles were administered via tail vein injection, and then mice were sacrificed after 24 h, and blood and tissues were collected. As shown in Figure 8, using both formulations, Phen circulating plasma levels and liver accumulation were significantly decreased compared to the free drug, while only Ph-NVs significantly increased Phen delivery into MB tissue (Figure 8b). Quantitatively, Ph-NV levels exhibited a 3.5-fold decrease in plasma and a 2-fold decrease in the liver while showing a 1.5-fold increase in medulloblastoma tissue, supporting their accumulation in cerebellar tumors. Quantitative real-time PCR showed that all formulations robustly decreased the intratumor levels of Gli1 and, to a lesser extent, of Ptch1, another Hedgehog (Hh) target gene (Figure 8c). Since Gli1 is a target of its own transcriptional activity, forming a positive feedback loop upon Hh pathway activation, while Ptch1 lacks such self-amplification, this mechanistic difference may underlie the differential degree of inhibition observed for Gli1 and Ptch1 mRNA in response to free or vehiculated Phen. Therefore, these data demonstrated that, compared to the free drug, Phen-loaded niosomes reduce liver accumulation of the biguanide, increase the delivery of the drug into intracranial MB, and significantly turn off oncogenic Hh signaling, thus representing a suitable tool to be tested for further therapeutic applications.

## 4. Discussion

In recent years, increasing attention has been devoted to drug repurposing for cancer therapy, together with the investigation of innovative nanocarriers aiming at improving the delivery of repurposed therapeutics [55]. In this paper, we have highlighted the potential of repurposing the antidiabetic drug Phen for MB treatment, combined with a novel vehiculation strategy to reduce its toxicity.

We have developed a novel, efficient amphiphilic-based vesicle approach to deliver Phen into cerebellar tumors, with a significant reduction of intrahepatic delivery (Figure 8).

Liposomes and niosomes have already been proven to be effective and safe delivery systems. Liposomes, in Doxil, have been on the market since 1995 [56], while niosomes were produced for the first time by the L’Oreal cosmetic industry in the 1970s [57], and then they have been widely studied since 1996 [58]. Here, Phen delivery via vesicular systems was demonstrated to be effective in terms of intracellular delivery, antiproliferative activity, target specificity and selective distribution within MB tissue. Ph-LVs inhibit intratumor Hh signaling similarly to free Phen. However, it is important to point out that the use of these nanoparticles reduces liver accumulation of biguanide compared to the free drug. This difference is relevant since the most concerning side effect of Phen is lactic acidosis, which is due to the inhibition of hepatic gluconeogenesis. Therefore, compared to free Phen, the use of Ph-LVs has the advantage of decreasing liver accumulation.

Niosomes also demonstrated enhanced targeting in animal models by increasing Phen delivery and efficacy within the cerebellar tumor. This enhanced activity can be attributed to several factors intrinsic to the niosomal delivery system. Firstly, niosomes, composed of nonionic surfactants and cholesterol, offer improved chemical and physical stability compared to liposomes, which are more susceptible to oxidative degradation and hydrolysis [59,60]. This improved stability likely results in more consistent drug release and better preservation of Phen pharmacological activity. Furthermore, niosomes have demonstrated superior ability to cross the BBB, a major limitation for drug delivery to brain tumors. Their surfactant-based structure facilitates enhanced permeability either by modulating tight junctions or through adsorptive-mediated endocytosis [61], which may increase Phen accumulation in tumor tissue. In addition, niosomes allow for efficient drug encapsulation and sustained release, which can enhance intracellular drug retention and cytotoxicity in target cells [62].

Together, these features are likely to contribute to the stronger inhibitory effect observed with niosomal Phen in our MB model. It will be relevant to assess the in vivo therapeutic efficacy of Phen-loaded vesicles in preclinical MB models. Phen has shown a significant therapeutic efficacy in SHH MB as well as in glioblastoma, another aggressive intracranial malignancy associated with poor prognosis [63], thereby representing an attractive candidate for drug repurposing in oncology. In addition, clinical trials are currently ongoing to evaluate the safety and efficacy of free Phen in cancer patients [54]. The possibility to use an optimized strategy to target the biguanide directly to the brain, reducing potentially harmful side effects, may thus represent a relevant further weapon to be evaluated in clinical settings. Further studies are needed to address this relevant issue.

## Figures and Tables

**Table 1:** Sample composition.

Samples	DMPC (mM)	Tween 20 (mM)	Chol (mM)	Phenformin (mM)
LVs	49	-	30	-
NVs	-	15	15	-
Ph-LVs	49	-	30	41
Ph-NVs	-	15	15	41

**Table 2:** Chemical–physical properties of the sample. Data are presented as means ± SD (*n* = 3).

Sample	Mean Diameter (nm) ± SD	ζ-Potential (mV) ± SD	PDI	Phenformin Concentration (mg/mL)
LVs	151 ± 1	−4 ± 1	0.20	-
NVs	190 ± 9	−19 ± 1	0.16	-
Ph-LVs	160 ± 3	−8 ± 1	0.27	5.2
Ph-NVs	191 ± 3	−17 ± 1	0.17	4.9

**Table 3:** Bilayer analysis. Examination of fluidity, polarity, and microviscosity values for Ph-LVs and Ph-NVs.

Sample	Polarity (I1/I3)	Microviscosity (IE/I3)	Fluidity (Anisotropy)
LVs	1.05	0.44	0.25
Ph-LVs	1.01	0.55	0.12
NVs	1.04	0.46	0.25
Ph-NVs	1.01	0.60	0.11

## Data Availability

The original contributions presented in this study are included in the article/Supplementary Material. Further inquiries can be directed to the corresponding authors.

## Supplementary Materials

The following supporting information can be downloaded at: https://www.mdpi.com/article/10.3390/nano16090566/s1, Figure S1: Raw images of Western blots related to Figure 5: (a) P-ACC, (b) ACC, (c) Vinculin from cell extracts shown in Figure 5c.

## Abbreviations

The following abbreviations are used in this manuscript:

MB	Medulloblastoma
Hh	Hedgehog
SHH	Sonic Hedgehog
Phen	Phenformin
PTCH	Patched
SMO	Smoothened
T2DM	Type-2 diabetes mellitus
LDHA	Lactate dehydrogenase
BBB	Blood–brain barrier
DMPC	1,2-Dimyristoyl-sn-glycero-3-phosphocholine
Tw20	Tween 20
Chol	Cholesterol
HS	Human serum
BS	Bovine serum
aCSF	Artificial cerebrospinal fluid
NV	Nano vesicle
LV	Lipid vesicle
CMC	Critical micelle concentration
DMEM	Dulbecco’s modified Eagle medium
SAG	Smoothened agonist
