# Supplementary material for: Brain-Targeted Delivery of Phenformin Using Phospholipid and Non-Phospholipid Vesicles for SHH Medulloblastoma
Source: Nanomaterials (Basel). 2026 May 4;16(9):566. doi: 10.3390/nano16090566 (PMC13164784; doi:10.3390/nano16090566)
Supplement: Supplementary file 1 [file nanomaterials-16-00566-s001.zip › nanomaterials-4199826-supplementary.pdf]

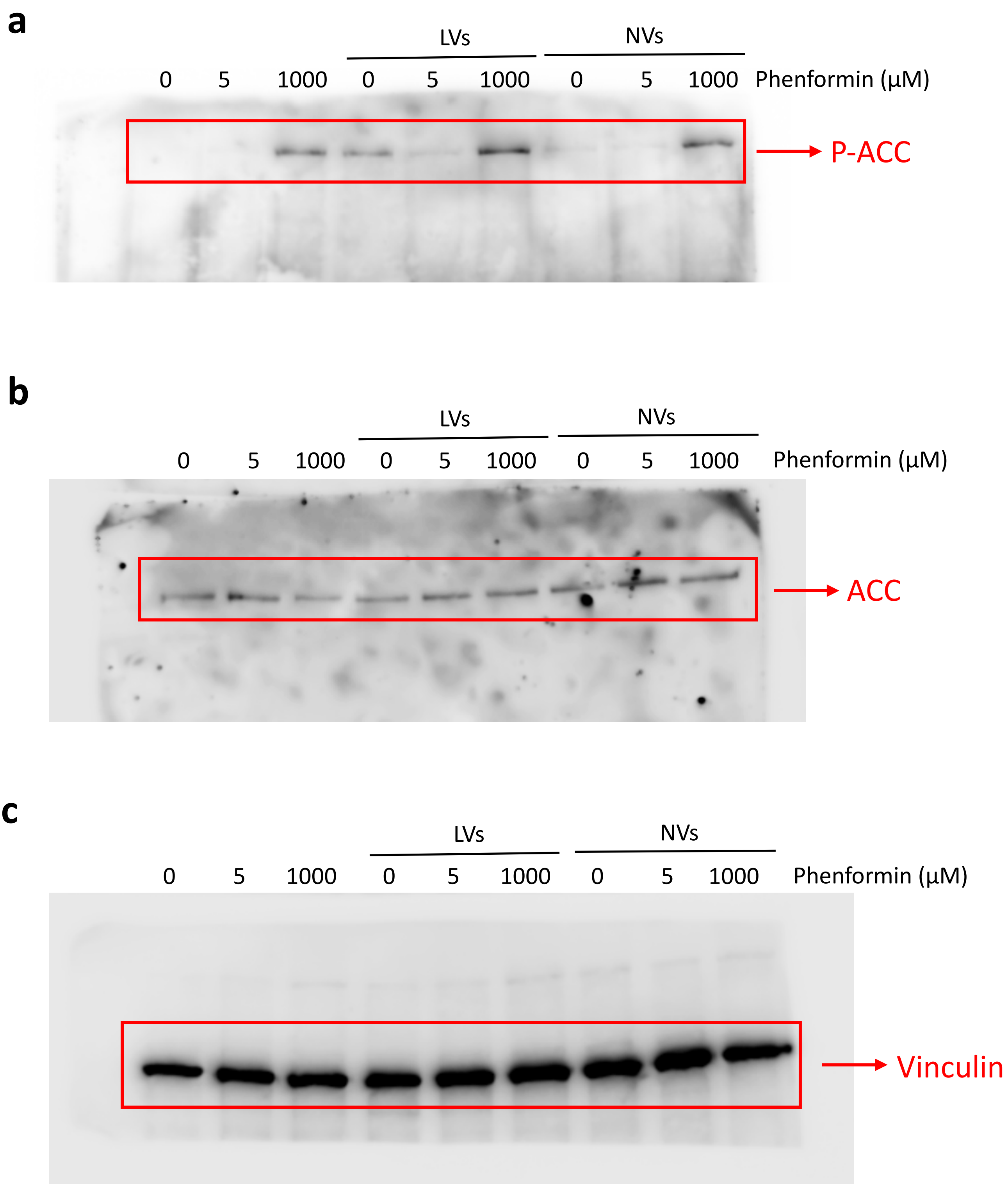

Supplementary Figure 1. Raw images of western blots related to Figure 5: (a) P-ACC. (b) ACC, (c) Vinculin from cell extracts shown Figure 5 (c).
